# Advancing the psychometrics of reverse-keyed items: enriching cognitive theory by a logical and linguistic perspective

**DOI:** 10.3389/fpsyg.2025.1684612

**Published:** 2025-10-20

**Authors:** David Elek, Hynek Cígler, David J. Grüning, Stanislav Ježek

**Affiliations:** ^1^Psychology Research Institute, Faculty of Social Studies, Masaryk University, Brno, Czechia; ^2^GESIS – Leibniz Institute for the Social Sciences, Mannheim, Germany; ^3^Department of Psychology, Heidelberg University, Heidelberg, Germany

**Keywords:** reverse-keying, reverse-coding, method factor, cognitive processing, logic, linguistic, polarity effect, negation

## Abstract

Inclusion of reverse-keyed items in a questionnaire usually impacts its factor structure and reliability. Therefore, their presence or absence also affects measurement validity, yet a clear consensus on their use is missing. In this paper, we provide an overview of the literature on the use of reverse-keyed items. We outline the typical arguments for and against their use, along with the cognitive explanatory framework commonly used to account for the associated issues. We further argue that while the cognitive model of responding is theoretically meaningful, it cannot, on its own, identify specific error sources from reverse-keyed item sets, and that issue lies in the implicit assumption regarding how reverse-keyed items should function. Furthermore, we note that literature on reverse-keyed items is at an impasse, with conflicting recommendations and inconclusive results. As a solution, we introduce a logical and linguistic perspective to advance our understanding of reverse-keyed items. This perspective allows researchers to understand that response inconsistencies in a statistical model do not necessarily indicate logically inconsistent answers from the respondent. Enriching the cognitive model with a linguistic perspective, which has been missing in psychometric literature, allows us to differentiate between mere statistical and actual response inconsistency. Based on this combination of cognitive and linguistic theory, we advance the historical analysis of response bias by suggesting that future research should closely draw from linguistic concepts to arrive at a promising explanatory framework that can then better inform modeling decisions. However, further empirical studies are needed to test our hypotheses and evaluate the magnitude and relevance of the proposed linguistic effects.

## 1 Introduction

When constructing a self-report scale a choice presents itself to the researcher: should the so-called reverse-keyed items be included? How will the validity of my instrument be affected? Should I risk response biases related to social desirability and acquiescence bias by omitting reverse-keyed items? Or should I include them, but risk an increased cognitive burden on my respondents, lowering the scale reliability and making the factor structure more complicated?

Psychometric and survey methodology literature is replete with arguments, recommendations, and best practices when it comes to using reverse-keyed items, but alas, no consensus has been achieved so far (Kam, 2018). We firmly believe that the reason for this impasse is the lack of a coherent explanatory framework due to the neglect of linguistics. As a result, the majority of research concerning reverse-keyed items is inconclusive and does not, in its current form, help advance psychometric knowledge when it comes to using reverse-keyed items. In this paper, we follow from Kam’s (2018) observation and build on the first steps taken in a similar direction (Kam, 2023; Kam et al., 2021; Kam and Meyer, 2022) to arrive at a more complete explanation of why reverse-keyed items prove to be so troublesome (yet worth including).

We argue that linguistic properties of item formulations introduce a systematic and largely overlooked source of variance that may masquerade as poor reliability or multidimensionality, when in fact the issue lies in linguistic or logical ambiguity. Consequently, traditional psychometric models may misattribute these effects to respondent inconsistency, thereby compromising the interpretability of latent constructs. To advance the validity of measurement instruments, particularly in clinical and applied settings, it is essential to integrate linguistic theory into our models of the response process.

It is important to emphasize that reverse-keyed items are not uncommon in clinical assessment. For example, the State–Trait Anxiety Inventory (STAI; Spielberger, 1983) explicitly includes several reverse-keyed items. In contrast, the Beck Depression Inventory-II (BDI-II; Beck et al., 1996) does not employ reverse scoring directly, yet several items are formulated using linguistic negation (e.g., “I do not enjoy things anymore”), which may still introduce cognitive or interpretative complexity.

We will restrict our focus to Likert-type items, as they are the most used and most researched type of item format in regard to reverse-keyed items. Most of our arguments will also hold for other self-report item formats that are based on (dis)agreement with a presented statement or selecting from several statement (as in BDI-II). However, we would like to make it clear that the effects we propose warrant further empirical testing; our article is intended as a call for both applied and theoretical researchers to expand their perspective when it comes to reverse-coded items. It is not a definitive statement of which effects are present and which are not. As a result of the novelty of our proposed approach, we cannot provide direct empirical evidence for our hypotheses. However, we believe that the linguistic perspective offers a promising direction for future research and that its utility should be examined in empirical studies.

## 2 The problem of reverse-keyed items

Reverse-keyed (or reverse-coded) items are statements that are related negatively to the measured construct. As such, agreement with these items should place the respondent on the negative pole of the measured construct (even if the said pole is only implied, i.e., having less of the measured construct). Items may be reversed due to their content (mentioning a manifestation of lower levels of construct), due to their wording (usually using negations), or (rarely) due to a reversed response scale. Originally, Likert (1932, p. 46) suggested that half of the scale should refer to the opposite pole. That is, the respondent at one pole of the attitude being measured should answer in agreement with only half of the items and in disagreement with the rest. In this way, it is possible to distinguish respondents who answer “honestly” from those who agree with any given statement regardless of its content.

The use of reverse-keyed items has subsequently become widespread as a method for controlling response biases, such as inattentive or acquiescent responding. This is because reverse-keyed items automatically average out scores of respondents who choose only one type of response across all items, preventing them from achieving an upwardly biased total scale score (Baumgartner and Steenkamp, 2001; Furr, 2011, p. 23). Another reason why reverse-keyed items might help control for response biases is that their (negative) formulation serves as a “cognitive speedbump,” alerting respondents and causing them to respond in a more controlled, less inattentive manner (Podsakoff et al., 2003).

Outside of their use as a means to control response biases, reverse-keyed items also serve to improve the content validity of a given scale by better covering the entire continuum of the measured construct (Tay and Jebb, 2018; Weijters and Baumgartner, 2012). Using just positively keyed (or regular) items would only allow us to differentiate between people at the higher end of the construct’s level and everyone else. Typically, disagreement with a positively keyed item is due to one of two reasons. Either the respondent disagrees because they are at the opposite end of the scale (e.g., an introverted respondent disagreeing with an extraversion item) or they are simply not as far up the scale as the item is (e.g., average respondent). The second reason is particularly notable, because Likert scales generally employ strongly worded items (in either direction), but not moderately worded items, as those exhibit undesirable properties, such as lower inter-item correlations, lower reliability and increased scale dimensionality (Tay and Jebb, 2018; Tay and Kuykendall, 2017).

Do note, however, that this does not necessarily affect every positively keyed item, as it depends on how extremely worded the item is in relation to the whole scale. The point is that by excluding reverse-keyed items from a scale, we rob ourselves of the opportunity to distinguish between the reasons for disagreement with the positive-keyed items. Unsurprisingly, doing so can then often bias the measured construct’s relationship with other validation criteria (Kam and Meyer, 2015).

Some constructs also might be more suited to assessment via positively keyed items, while others by using reverse-keyed ones. A good example is Relationship Structures Questionnaire (ECR-RS; Fraley et al., 2006), using nine items to measure two dimensions: attachment anxiety and avoidance. While all three anxiety items are positively keyed, four of six avoidance items are reverse-keyed (lower scores represent secure attachment). Obviously, it is much easier to generate “non-avoidance,” and thus reverse-keyed items. For example, changing an item “It helps to turn to this person in times of need” into a regular (i.e., avoidant) one while preserving its wording clarity could prove difficult. On the other hand, anxiety is easier to measure directly with positively keyed items, for example: “I often worry that this person does not really care for me.”

### 2.1 Negative consequences and their possible causes

However, reverse-keyed items are not without their downsides. It is common to observe reduced internal consistency and the emergence of multidimensionality in a scale consisting of both positively and reverse-keyed items (Bulut and Bulut, 2022; Chyung et al., 2018; Schriesheim et al., 1991; Swain et al., 2008; Weijters and Baumgartner, 2012; Zeng et al., 2020). In particular, the emergence of multidimensionality can be problematic, as it raises the question of whether the dimensionality of a construct differs from what was expected (note that this question does not disappear if reverse-keyed items are omitted; it just remains hidden). Generally, these observations are attributed to “inconsistent responses” on the part of respondents when responding to reverse-keyed items due to the basic assumption that positively and reverse-keyed items are more or less equivalent indicators of the measured construct (Chyung et al., 2018). Consequently, any effects related to keying are to be treated as method effects (Weijters et al., 2013), often referred to as the “item wording effect,” “polarity effect,” or “reversal effect” (Kam, 2018). As such, a number of publications (e.g., Menold, 2020; Swain et al., 2008; Weijters et al., 2013; Weijters and Baumgartner, 2012) have attempted to provide an explanation for what causes respondents to answer inconsistently on a reverse-keyed item. These can be summarized into three causes:

First, insufficient motivation or cognitive effort when responding to reverse-keyed items. This can take the form of inattention to the negating particle (van Sonderen et al., 2013), where the respondent misses the fact that the item is reverse-keyed and responds as if it were positively keyed. Similarly, insufficient motivation and/or acquiescent responding can lead to respondents answering positively to affirmative and negatively to negated statements (Weijters and Baumgartner, 2012).

Second, a difficulty with verification and mapping of a response. In case of item verification difficulty (Swain et al., 2008), respondents have difficulties verifying a negated statement as compared to affirming statements. Similarly, even if the respondent manages to verify the negated statement, the cognitive load of doing so can lead them to mistakenly reverse their intended response when selecting a response option. For example, take an item “I do not like being center of attention” and a respondent first verifying if they do like being center of attention and answering affirmatively, forgetting the fact the item is reverse-keyed.

Lastly, a misinterpretation of the reversal. Conceptualized as reversal ambiguity (Weijters and Baumgartner, 2012), where the respondent accurately attends to the content of the item but does not understand the displayed antonym as the (direct) opposite of its positively keyed word also used in the scale. This is not necessarily an error on part of the respondent, but that hinges on the fact whenever the chosen antonym can be logically considered an opposite to the adjective in the positively keyed item.

These explanations, often placed within the cognitive response process model (Cognitive Aspects of Survey Methodology, CASM; Tourangeau, 2018), run into issues when it comes to empirical testing. Most of the time, they are assessed indirectly, either using an index that should capture a given response process (e.g., acquiescent responding: Menold, 2020; Weijters et al., 2013) or using more flexible item response models (Cole et al., 2019). Alternatively, in Structural Equation Models (SEM) by typically including some sort of method factor(s) for the reverse-keyed items (Kulas et al., 2019; Schmalbach et al., 2021; Tang et al., 2024; Weijters et al., 2013). The method factor is then related to some external criteria (e.g., a social desirability scale) or has specific constraints placed on it based on the researcher’s conceptualization of a given misresponse cause. At best, integration of eye tracking (Baumgartner et al., 2018; Koutsogiorgi and Michaelides, 2022) offers valuable information on which parts of the items respondents fixated on.

Unfortunately, all these approaches run into the same issue. At best they can identify the presence of misresponses, but they cannot support the claims as to their origin. The reason is that they need to employ a measurement model to identify those inconsistent responses in the first place. And as we have noted earlier, using reverse-keyed items hinges on the assumption that they work roughly the same as the positively keyed ones, just in the opposite direction (Chyung et al., 2018; Weijters and Baumgartner, 2012).

This assumption is thus built into modeling of method factors via SEM (or Item Response Theory, IRT), as the models used assume that items and the latent variable are related by the same link function (linear, logistic etc.). This means that by its very nature, it is not possible to identify misresponses and thus model method factors without first assuming that reverse-keyed items relate to the measured construct in the same manner as the positively keyed ones. In other words, what all these studies find are responses that are *inconsistent with the measurement model*, while trying to tack on an explanation invoking cognitive processes as to why these responses happen in the first place. It is surprising because one can find mentions in the literature of this assumption being potentially problematic (Chyung et al., 2018; Weijters and Baumgartner, 2012), but no one seems to ask themselves, if the problem does not lie in measurement models that are based on it. Inconsistent responses are framed as by definition problematic, without sufficient attention to whether they cannot be logical, after all.

### 2.2 Are the responses truly inconsistent?

Before we address the issue of whether inconsistent responses are truly inconsistent, we have to provide a more nuanced definition of reverse-keyed items. We have previously alluded to the possibility of creating reverse-keyed items in several different ways. However, with a few exceptions, reverse-keyed items are treated as a homogeneous category in the literature, that is, there are no conceptual distinctions between different types of reverse keying (Weijters and Baumgartner, 2012). Unfortunately, different kinds of reversing may cause different effects, as we show later. Therefore, we will first provide a basic outline of these reverse-keyed item types in this section.

The process of reverse-keying (i.e., restructuring an item to have opposite poles) can be achieved in more than one way. The first is through negation of a statement (e.g., “I am happy.”). Negation of a statement can take many forms. Commonly, the verb or adjective is negated directly (“I am *un*happy” or “I am *not* happy”), but less so through negation of an adverb (“I am *not very* happy.”), as the latter restricts the range of possible answers (i.e., a respondent indicating being not very happy at all can be either feeling neutral or feeling very unhappy). Negation of a statement can also be done by using an antonym,^1^ fully substituting the adjective used:

“I am *happy*.” → “I am *sad*.”

Antonyms can again be distinguished into morphological and lexical (Aina et al., 2019). Morphological antonyms are formed by affixal negation, that is, a negation added as a prefix to the positional form of the adjective:

capable → *in*capable

Conversely, lexical antonyms have a different root than their positive article:

alive → *dead*

Some adjectives have antonyms in both morphological and lexical forms (see the example of “happy”), while some have no meaningful morphological opposite (e.g., inert – ert, awful – awless.)^2^. For the sake of simplicity, we will distinguish negations and antonyms for reverse-keyed items as follows. To summarize, negation is formed through either the negation of a preposition (typically a verb) or an adverb (typically a frequency) in the item statement. Antonyms are either morphological or lexical, formed exclusively through a change of the attribute in the statement (almost always an adjective).

Reverse-keyed items can, thus, be classified according to being constructed by using negation or antonymy (Schriesheim et al., 1991). This division can be displayed in a two-by-two format visualization (Weijters and Baumgartner, 2012), as shown in Table 1. Note that the difference between regular and antonym is completely arbitrary. In the case depicted in Table 1, the measured construct is “human height” or “tallness.” If “shortness” were measured instead, “I am tall” would be an antonym (and vice versa). If we go into a detail, markedness (Andersen, 1989) plays a role. The adverb “tall” is considered to be unmarked (typical pole of a scale) compared to marked “short.” For instance, negative adjectives according to the markedness criterion carry an additional presupposition compared to their positive counterparts (Rett, 2015, p. 49). An item such as “I am as short as my colleagues at work” would have the interpretation that both the speaker and the colleagues are short. However, “I am as tall as my colleagues at work” does not presuppose that “tall” applies to both. In fact, the sentence is compatible with all of them being short. Therefore, we consider markedness to be a fruitful area of research in reverse-keyed items’ problem.

**Table 1:** Classification of items according to the presence of negation and antonyms.

	Negation absent	Negation present
Antonym absent	Regular: *“I am tall.”*	Negation: *I am not tall.”*
Antonym present	Antonym: *“I am small.”*	Negated antonym: *“I am not small.”*

Regular and negated antonym items are both positively keyed while antonym and negation are reverse-keyed.

Let us now consider an example of two self-report items measuring human height: “I am tall” and “I am short.” The main assumption regarding reverse-keyed items is that they measure the same entity as the positively keyed item, only with reversed polarity (Weijters and Baumgartner, 2012).

That is, if respondents agree with a positively keyed item (i.e., regular item or negated antonym), they should logically disagree with an item referring to the opposite pole (i.e., negated item or antonym). But is this necessarily a true assumption? We have already mentioned that so-called reversal ambiguity is not by definition a source of error. In fact, under certain circumstances, it can be logical for respondents to disagree with both the positively and reverse-keyed items (Weijters and Baumgartner, 2012). A prime illustration of this phenomenon is presented by Kam et al. (2021) who asked respondents to indicate their physical height. Respondents with above-average and below-average height would respond consistently. That is, respondents with above-average height would agree with regular items and negated antonyms, and disagree with negated and antonymic items, and respondents with below-average height would do the opposite. However, respondents of average height would disagree with both regular and antonymic items and agree with both negated items and negated antonyms. The full pattern is shown in Table 2.

**Table 2:** Plausible response patterns for each item variant.

Position of respondent on the scale	Regular item	Negation	Antonym	Negated antonym
	*“I am tall.”*	*“I am not tall.”*	*“I am short.”*	*“I am not short.”*
Above-average	Agreement	Disagreement	Disagreement	Agreement
Average	Disagreement	Agreement	Disagreement	Agreement
Below-average	Disagreement	Agreement	Agreement	Disagreement

Adapted from Kam et al. (2021).

**Figure 1:**
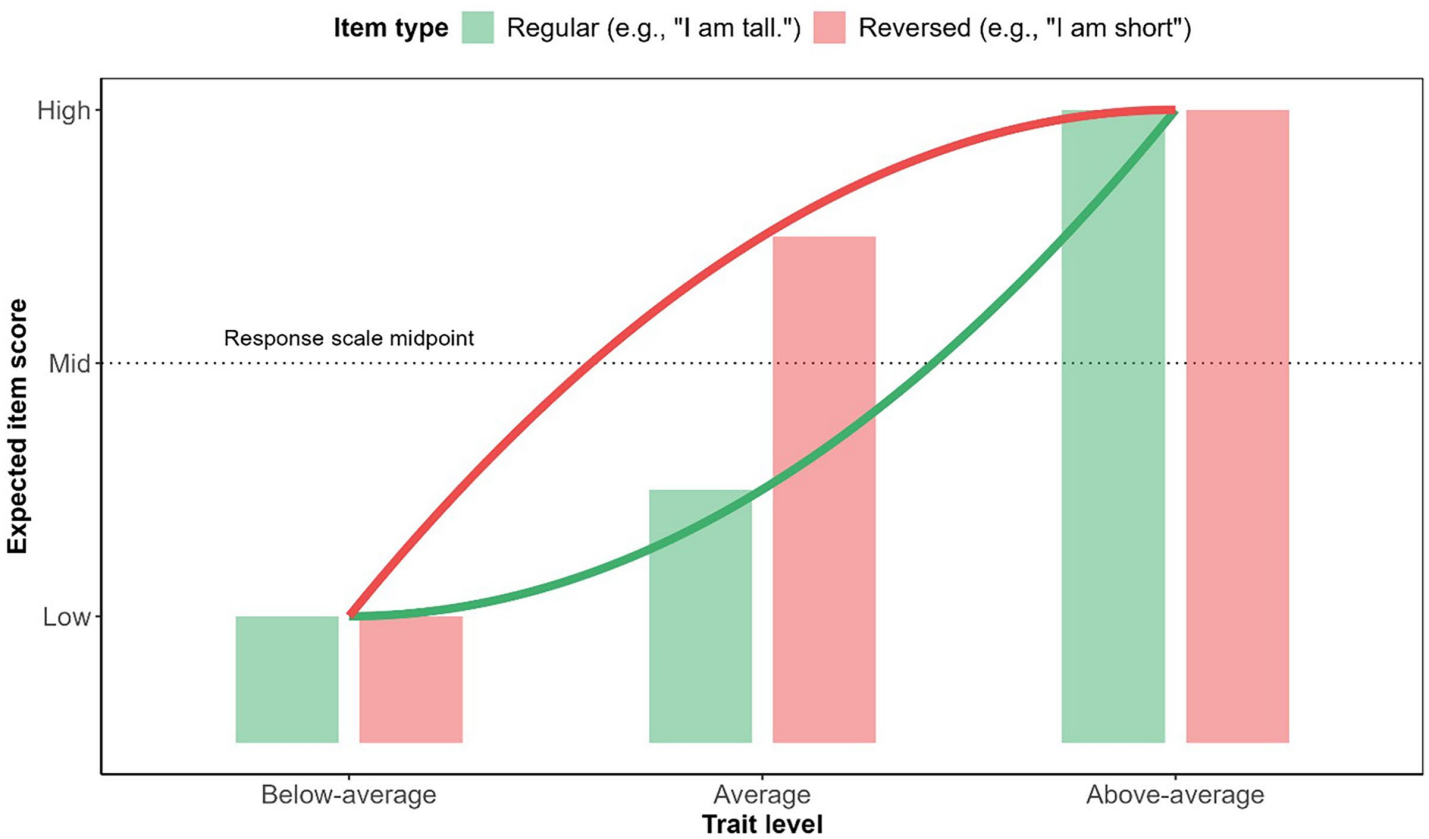
Expected responses for below-, above- and average respondents. Reprinted with permission from Rečka et al. (2025).

Kam et al. (2021) further argue and analytically demonstrate that the substantial response inconsistency of respondents of average height is the main source of multidimensionality in factor analysis. Specifically, the single-factor model (i.e., here of physical height) accurately describes responses of participants with attributes closer to the extremes (i.e., being very tall or short). However, this model is increasingly unable to model non-extreme, average expressions of the measured attribute. This is because the linear factor model assumes a linear and monotonic relationship between the latent variable and the items. As can be seen from Table 2, average respondents break this monotonic trend. A visualization of this effect can be seen in Figure 1. The consequences are then similar to the well-known “difficulty factor” in achievement tests (McDonald and Ahlawat, 1974).

This insight leads to Kam et al. (2021) questioning the widely held belief that responses that seem inconsistent (i.e., here the average-height respondents) point to an evaluation error. Quite the opposite can be true: Respondents might answer in a perfectly logically consistent manner, but inconsistent with what is predicted by a given psychometric model.

In a follow-up study Kam and Meyer (2022) demonstrate that the factor for the positively-keyed items and reverse-keyed items are nonlinearly related. Moreover, our research suggests that positively and reverse-keyed items systematically differ in their relationship to the measured construct (Rečka et al., 2025), which would cause their own factors to have non-linear relationships that Kam and Meyer (2022) observed. Moreover, if a non-linear measurement model is used (such as an ordinal factor analysis) instead of a linear model (such as a traditional continuous factor analysis), the problem is significantly reduced (Rečka et al., 2025). We can hypothesize that if a more flexible model with a better fit to the data were used, then spurious multidimensionality would disappear completely.

However, from the perspective of response process validity, treating mismatches between observed and expected responses as evidence of respondent error or bias overlooks the linguistic complexity inherent in item formulation. In this light, the reverse-keyed items method factor cannot be readily understood as a “real” individual characteristic, such as the tendency to agree, but merely as the variance of responses that is inconsistent with the proposed statistical model. This also means that finding inconsistency between the data and the statistical model does not necessarily imply a logical inconsistency of the responses or points to cognitive errors made by a respondent. At the same time, from this logical perspective, factor models cannot exclude possible response errors either. Respondents may answer inconsistently with the statistical model because it is logically plausible or because they actually made a mistake due to motivation or inattention. In conclusion, it is problematic to assume *a priori* that all model inconsistencies point to actual response biases, as is common in the literature on reverse-keyed items (Kam et al., 2021). Rorer (1965) already pointed out that interpreting inconsistent responses between positively and reverse-keyed items as evidence for the existence of response styles is a misguided idea, especially so when the oppositely keyed items in question do not present logically exclusive statements. According to the logical perspective on what the misfit between data and model cannot inform about, it is evident that an additional examination of logical consistency between positively and reverse-keyed items is needed. Linguistics offers a useful perspective.

### 2.3 The linguistic perspective

In an earlier section, we described a typology of reverse-keyed items depending on their mode of reversal. We distinguished between reversal by negating the target word or by using one of its antonyms. From a linguistic perspective, there exist several caveats when using antonyms to reverse an item’s meaning. First, let us revisit the case of reversal ambiguity (Weijters and Baumgartner, 2012), where an antonym is not necessarily interpreted by the respondents as the opposite of the target word. To understand this issue, we have to understand the two types of opposition that can be found in logic (Horn, 2020), namely, *contrariety* and *contradiction*. When using antonyms, a case of contrariety means that the word and its antonym are mutually incompatible but not mutually exclusive. That is, a respondent can indicate disagreement on both at the same time without being contradictory. The classic example for this is the pair “small” and “large.” It is not possible to be both small and large at the same time, but it is possible to be neither small nor large. Cases of contradiction do not allow for these instances of indifference. Here the word and its antonym are mutually exclusive. The classic example for this case is the pair “alive” and “dead.” It is impossible to be both alive and dead, but it is also not possible to be neither alive nor dead.

These two types of opposition (i.e., contrariety and contradiction) are commonly also conceptualized by the antonym’s boundedness to the target word (Paradis and Willners, 2006). Boundedness of an antonym refers to whether or not the negation of a word directly corresponds to its antonym. *Unbounded* antonyms express a range of a scale (e.g., “wide” and “narrow”) and are counter-directional (Paradis and Willners, 2006, p. 2), that is when they are intensified (e.g., *extremely* narrow) they move away from another on the scale. Furthermore, they do not reach the end of the scale, as their scale is considered unbounded. Conversely, *bounded* antonyms express “either-or” relation (e.g., “alive” and “dead” from before). Despite that, bounded antonyms can be laid out on a scale, while at the same time establishing boundaries of such scale, and thus expression such as “neither alive nor dead” can be interpreted as either “almost dead” or “half alive” (Paradis and Willners, 2006, p. 3).

As we have outlined above, participants’ responses are not necessarily logically inconsistent when they disagree with both item statements that include an unbound target word and antonym (e.g., “small” and “large”). In contrast, disagreeing with two items if they include a bound pair of target word and antonym, respectively, is logically inconsistent, as the pair is logically contradictory. Importantly, agreeing with both items is logically inconsistent in both cases (i.e., unbound and bound). However, that only applies if the target word and its adjective occupy the same scale. Furthermore, in cases of two negated statements (“I am not tall” and “I am not small”), agreement with both is logically consistent as it implies the same as disagreement with both their negated versions. Unfortunately, researchers often miss this and assume that if the two statements stand in seeming opposition, that disagreement with one should imply agreement with other.

Antonyms can be also conceptualized according to their degree of canonicity (Paradis et al., 2009), namely, the degree to which an antonym and its regular target word can be considered a pair semantically and on the basis of its conventional use in language (by lay people). A highly canonical pair is more strongly anchored in memory and is more frequently used in language. Importantly, Paradis et al. (2009) argue and demonstrate that such highly canonical pairs of antonyms are relatively small in number in the language compared to pairs of medium and low canonicity. In other words, few pairs of antonyms can be accurately used to express real opposition. With decreasing canonicity, disagreement among people increases about which antonyms are real opposites of a particular target word. This insight has major implications for interpretation of responses on reverse-keyed items. Psychometricians may generate reverse-keyed items using antonyms in the belief that this covers both poles of the superordinate construct (Tay and Jebb, 2018; Weijters and Baumgartner, 2012). However, when using any but a canonical pair of antonyms (e.g., good-bad), the possibility increases substantially that respondents do not understand the two oppositely keyed items as true opposites, subsequently responding in a manner that is later interpreted as inconsistent responding.

We have already briefly touched on negation as a way to form reverse-keyed items, but it is also important to mention caveats of using negation for reverse-keyed items. While negations might seem like a tempting way to ensure that the negated item simply refers to the opposite direction of the regular one, there is large body of literature pointing to the fact that recalling and processing negative information is more cognitively demanding (Khorsheed et al., 2022; van Tiel et al., 2019; Van Tiel and Pankratz, 2021), negated statements harder to disagree on (Kamoen et al., 2017) and require more larger numbers of revisits (Koutsogiorgi and Michaelides, 2022). Moreover, these effects are stronger if an antonym occurs unexpectedly, “out-of-the-blue” (Kaup and Dudschig, 2020), which might have consequences if only a single (or a few) reverse-keyed items are presented in a questionnaire.

Reversed-keyed items are, therefore, more prone to errors due to both aforementioned cognitive load and due to the fact negations in natural language offer diverse pragmatic interpretations of a word. An example could be the finding that boundedness of antonyms is further moderated by the presence of negation in the used items (Paradis and Willners, 2006). Negation of the item content can either serve as a logical operator of the opposite (in the case of bounded antonyms) or as a weakening modifier (in the case of unbounded antonyms), where the negated unbound antonym is interpreted as a milder degree of the original antonym rather than the direct opposite (Paradis and Willners, 2006). Ironically, the negation of an adjective through its verb can also produce the opposite effect: The so-called “inference towards the antonym” (or “negative strengthening”; Ruytenbeek et al., 2017) describes respondents’ asymmetric inference towards antonyms of different valence, as follows:

1. “I am not big.” → “I am small.”
2. “I am not small.” ↛ “I am big.”

In addition to the antonym’s polarity, the effect of asymmetric inference is also stronger for morphological antonym pairs (Aina et al., 2019; Ruytenbeek et al., 2017). This variability in interpretation asymmetry has also major implications for the use of reverse-keyed items. In the construction of a scale, we might assume that we have generated pairs of items that refer to opposite poles of the scale. However, in practice, respondents might interpret only some of the chosen negated or antonymic items as polar opposites, while others are interpreted as only weakening the positively keyed item formulation or as referring more to the middle of the response scale.

As a result, whenever we elect to use antonyms or negations to form reverse-keyed items, we run the risk of mixed interpretations of items, resulting in responses that are highly inconsistent with the monotonic response tendency (i.e., the more/less I think to have a certain trait, the more/less I agree with the item) as predicted by the psychometric model used. While the above list of linguistic concepts is by no means exhaustive, it serves as an illustration of the complexity when dealing with negation and antonyms. Omitting linguistic theory and blending all the reversal types into a single class of “reverse-keyed items” with presumably similar characteristics may invalidate research results; especially if a small, non-representative sample of reverse-keyed items is studied within a few questionnaires. The reversal effect may be highly heterogenous across different types of negations and antonyms.

By this we do not intend to claim that the proposed linguistic perspective should replace existing cognitive response models (i.e., CASM), but rather that it offers a fruitful extension that can explain *why* and *how* reverse-coded item effects occur. We provide two examples.

First, markedness affects both the comprehension and retrieval steps of the response process. Comprehension depends on the presuppositions carried by an unmarked adjective and consequently influences what the respondent retrieves from memory. Markedness can therefore serve as a linguistic explanation for confirmation bias at retrieval (Weijters and Baumgartner, 2012) and for item verification difficulty at comprehension (Swain et al., 2008), particularly if the presupposition in the marked adjective runs contrary to the respondent’s experience (e.g., a tall respondent being asked whether he is as short as other people). Similarly, canonicity and boundedness can serve as explanations for reversal ambiguity at the comprehension stage (Weijters and Baumgartner, 2012) and for inconsistent responses to both positive and reverse-coded items—responses that are commonly attributed to respondent carelessness and/or acquiescence (Bulut and Bulut, 2022; Garrido et al., 2025). This is not to say that these linguistic effects cannot coexist with other explanations. For example, while there is a plausible linguistic explanation for why a respondent might agree with both a regular and a reverse-keyed item, a cognitive error—such as inattention—is equally plausible in the absence of additional empirical evidence. However, as we have argued throughout this article, it is misguided to interpret all inconsistent responses solely through the lens of cognitive biases and errors, when linguistics offers a range of alternative explanations that warrant further empirical testing.

An additional consideration concerns differences in negation across languages. Because different languages use different forms of negation with various effects on item interpretation, it is also possible that the wording effect varies across languages. The assumption that the wording effect is independent of language is unjustified and should be an important focus of future research.

### 2.4 Going forward

As the previous section showcased, neither negations nor antonyms are exempt from issues when it comes to responding to items containing either one. Unsurprisingly, one can find recommendations against employing reverse-keyed items of any kind (Menold, 2020; Suárez-Álvarez et al., 2018; van Sonderen et al., 2013), against employing negations of any kind when generating items (Koutsogiorgi and Michaelides, 2022; Swain et al., 2008; Weijters and Baumgartner, 2012), but also for (careful) inclusion of antonyms (Baumgartner et al., 2018). However, as we have argued in this article, there are two reasons for these conflicting recommendations. One is that researchers often do not explicitly consider multiple types of reverse-keyed items in their studies but selectively demonstrate issues with either negation of antonymy. The second is that there is a notable gap between empirical linguistic findings about negations (and antonyms) and psychometric practice, which leads to impoverished understanding of why item wording or reversal effects emerge in any given instrument. That is not so say that psychometricians do not offer various explanations for the reversal effects, but the connection between the purported cognitive processes and the actual modeling of the reversal effect via latent variable models is tenuous at best.

A cynical reader might conclude that reverse-keyed items are more trouble than they are worth. We emphatically disagree, in line with Kam (2018) and Weijters and Baumgartner (2012), as reverse-keyed items provide a key increase in content validity that cannot be easily substituted, even if one is skeptical of their capability to control for response biases. Moreover, as we already pointed out, the problem with reversals does not disappear if reverse-keyed items are removed. What if the validity impairing effect is not associated solely with reverse-keyed items, but also with the positive ones? Then, if we only keep positively keyed items in a scale, the reversal effect is still present, though we do not observe it and cannot control it.

Overall, what is needed is proper classification of reverse-keyed items, as even the simple Negation × Antonym framework we used in this paper does not sufficiently capture the complexity. Furthermore, an empirical investigation into the various linguistic concepts such as negative strengthening, canonicity and boundedness of antonyms is needed to ascertain their relevance and impact on responding and subsequent psychometric modelling. The most obvious point is that very often, the chosen antonyms are not mutually exclusive (contradictory) to the adjectives used in their sibling positively keyed item. Likewise, negating an adjective does not automatically lead to interpretation that would land at the other end of the scale. Rather than modelling latent heterogeneity in responses (Arias et al., 2020; García-Batista et al., 2021; Ponce et al., 2022) in an attempt to identify inconsistent respondents in and explain their inconsistent responses as due to (low) cognitive abilities or personality traits (Chen et al., 2024; Steinmann et al., 2022), we should first attend to how people interpret the language we choose in our items. A sufficiently granular classification of reverse-keyed items should be the first step in untangling the various conditions and interactions. Only after this empirical and theoretical base has been sufficiently established can we move towards the question of how to model these effects.

## 3 Conclusion

Our findings could have major implications for measurement validity in psychological research. Without a linguistically informed framework, reverse-keyed items may distort our thinking about the construct being measured and undermine both structural and criterion validity. What may appear as inconsistency or bias in the data may, in fact, may stem from linguistic ambiguity in item formulation rather than from respondent error. Importantly, this can occur regardless of whether reverse-keyed items are included or excluded from the instrument.

Even though psychometric and methodological literature has to some degree referenced linguistic work, we believe it is not nearly enough, and it is still marred by the assumption of reverse-keyed items functioning akin to a *mirror* to the positively keyed ones. The linguistic perspective shows that this is at best conditional, and often inaccurate. Without addressing these linguistic dimensions, psychometric models risk misattributing variance and mischaracterizing the cognitive mechanisms underlying item responses. By this we do not make the claim that various response biases and styles (i.e., acquiescence, inattention) do not exist and do not influence responses, rather that we should first rule out whether our models or assumptions might be incorrect. Specifically, even recent studies still approach the issue of reverse-coded items with the *mirror assumption* (e.g., Garrido et al., 2025; Steinmann et al., 2024; Zeng et al., 2024) and at best arrive at the detection of wording effects, their consequences for reliability and dimensionality, and possible covariates. In our view, this is a far cry from an explanation of *why* and *how* these effects occur—unless, of course, one considers the categorization of respondents into “consistent” and “inconsistent” (e.g., Chen et al., 2024; Ponce et al., 2022; Steinmann et al., 2022) sufficient as an explanation. Conversely, studies that utilize the CASM framework, typically through eye-tracking (e.g., Baumgartner et al., 2018; Koutsogiorgi and Michaelides, 2022), offer explanations for the cognitive sources of inconsistency, such as longer eye fixations indicating processing difficulties with negations. Yet in our view, they do not fully account for alternative explanations that do not involve cognitive error.

We thus believe that linguistics offers a promising way to expand our knowledge of reverse-keyed items, how to use them and how to model their responses. However, there is limited empirical support for most of our claims, which are primarily rooted in linguistic theory. Our assumptions have to be tested in future empirical studies. One possible design could be an explanatory systematic review focusing on differences across studies in the wording effect related to different types of reversals used in particular questionnaires. Another design would be an experimental study manipulating different types of reversals. Furthermore, due to the language dependency of at least some of the proposed effects, the proposed linguistic perspective is especially relevant for cross-cultural research, particularly when it comes to instrument translation and validation. Different grammatical forms of negations across languages can thus result in variations of the wording effect on the same scale. As a result, better understanding of how negation is interpreted across languages appears to be a critical piece of knowledge for cross-cultural research and scale validation.

We urge test developers and applied researchers—particularly in clinical settings—to re-evaluate the assumptions underlying their use of reverse-keyed items, considering the linguistic properties discussed here as possible contributors to measurement error. However, as the linguistic aspects of wording effect has not been studied yet, a clear guidelines for scale development cannot be provided. We stress that recommendations without considering the linguistic aspects could be misleading. Addressing linguistic issues related to reverse-keyed items is crucial for advancement in measurement in social sciences. According to our experience, up to 10% of systematic covariances across items in mixed-format scales may be related to reversals. The lack of knowledge as to “why” this effect occurs (and “with which reversals”) heavily undermines our reasoning about the measurement validity, and can lead to suboptimal or even erroneous decision related to item construction and selection with a direct impact on a scale’s validity. The psychometric answer to the wording effect cannot be based solely on implementing atheoretical psychometric models and identifying spurious factors but must be firmly grounded in linguistic theory; not least because language is very flexible in interpretation of negation, making it likely that the related reversal effects will be similarly varied.

## Footnotes

^1^Logically, antonyms are a subcategory of negation (Horn, 2020), but we will treat them as a unique category in the present paper, as was custom in other papers (e.g., Weijters and Baumgartner, 2012).

^2^We have not been able to find an example of an adjective that does not have at least a lexical counterpart.
